# Serum SAMD9 as a Potential Prognostic Biomarker for Pancreatic Cancer

**DOI:** 10.3390/cancers18172781

**Published:** 2026-08-27

**Authors:** Xingyu He, Ying Zheng, Xunjie Cao, Jianhao Zhang, Yang Yang, Qing Zhu

**Affiliations:** Department of Abdominal Oncology, West China Hospital of Sichuan University, Chengdu 610041, China; 2024224020221@stu.scu.edu.cn (X.H.); 2025324020140@stu.scu.edu.cn (Y.Z.); 2023224020153@stu.scu.edu.cn (X.C.); 2023324025340@stu.scu.edu.cn (J.Z.); 2022324020107@stu.scu.edu.cn (Y.Y.)

**Keywords:** pancreatic cancer, biomarker, SAMD9, diagnosis, prognosis

## Abstract

**Background:** Pancreatic cancer (PC) is one of the most aggressive malignancies and remains a leading cause of cancer-related mortality worldwide. Despite advances in diagnosis and treatment, reliable biomarkers for detection and prognostic assessment remain limited. Sterile alpha motif domain containing 9 (*SAMD9*) has been implicated in several human malignancies, but its clinical significance in PC has not been fully elucidated. Therefore, this study aimed to investigate the diagnostic and prognostic value of SAMD9 in PC. **Methods:** *SAMD9* expression was analyzed using transcriptomic data from the TCGA, GTEx, and GEO databases. Kaplan–Meier survival analysis, Cox regression, and receiver operating characteristic (ROC) curve analysis were performed to evaluate its prognostic and diagnostic value. Serum SAMD9 levels were measured by ELISA in 89 patients with PC, 55 patients with benign pancreatic diseases, and 47 healthy controls. **Results:** *SAMD9* expression was significantly elevated in PC tissues and was associated with unfavorable survival. Multivariate Cox regression identified *SAMD9* as an independent prognostic factor (HR = 1.704, 95% CI: 1.110–2.617, *p* < 0.05). ROC analysis demonstrated good diagnostic performance across multiple public cohorts, with AUC values ranging from 0.834 to 0.977. Serum SAMD9 levels were significantly higher in patients with PC than in the control groups (*p* < 0.05). Patients with high SAMD9 expression also had significantly shorter overall survival. **Conclusions:** SAMD9 is aberrantly expressed in PC and shows potential as a prognostic biomarker.

## Simple Summary

Pancreatic cancer remains one of the deadliest malignancies, and effective biomarkers for its detection and prognosis are still lacking. In this study, *SAMD9* was consistently overexpressed in pancreatic cancer tissues and was associated with poor patient outcomes. Analyses of public datasets showed that *SAMD9* expression had diagnostic and prognostic value in pancreatic cancer. However, validation in clinical serum samples showed that serum SAMD9 had only limited diagnostic performance, although it remained associated with patient prognosis. Overall, these findings support the potential prognostic relevance of SAMD9, while the diagnostic utility of serum SAMD9 requires further validation.

## 1. Introduction

Pancreatic cancer (PC) is a highly aggressive malignancy, with an approximately 13% 5-year survival rate and a median overall survival of less than one year [1]. It remains a major contributor to cancer-related mortality on a global scale [2]. Because of its subtle onset and nonspecific clinical presentation, pancreatic cancer is often detected only after the disease has progressed to an advanced stage, and only 10–20% are eligible for surgical resection [3,4]. Therefore, improving the diagnosis and prognostic evaluation of PC remains a major clinical challenge. Currently, carbohydrate antigen 19-9 (CA19-9) is the most widely used serum biomarker for PC [5,6]. However, its diagnostic accuracy is limited by insufficient sensitivity and specificity [7,8]. Owing to their convenience, minimal invasiveness, and cost-effectiveness, serum biomarkers are considered promising tools for the detection and clinical management of PC [9]. Consequently, the identification of novel serum biomarkers with higher diagnostic accuracy and prognostic value is urgently needed.

Sterile alpha motif domain containing 9 (*SAMD9*), encoded by the *SAMD9* gene located on chromosome 7q21.2, is an interferon-inducible protein involved in innate immune defense, inflammatory regulation, and cellular stress responses [10,11,12,13,14]. It functions downstream of tumor necrosis factor-α (TNF-α) and interferon-γ (IFN-γ) signaling pathways [13,15]. Research indicates that *SAMD9* plays a critical role in tumor development and progression. However, its biological functions appear to be tumor-type dependent. For example, increased *SAMD9* expression has been associated with postoperative recurrence and poor prognosis in esophageal squamous cell carcinoma and has been reported to promote glioma progression [16,17,18,19], whereas it exhibits tumor-suppressive effects in non-small cell lung cancer [20]. These findings indicate that the role of *SAMD9* varies considerably across different malignancies.

This study aimed to comprehensively evaluate *SAMD9* expression in PC using data from TCGA, GTEx and GEO databases and clinical serum samples, and to investigate its association with clinicopathological characteristics, diagnostic performance, and patient prognosis. Our findings may provide evidence supporting SAMD9 as a novel diagnostic and prognostic biomarker for PC.

## 2. Materials and Methods

### 2.1. Sample Information

This study was carried out at West China Hospital of Sichuan University (Chengdu, China), from March 2020 to July 2023. The research protocol received approval from the Ethics Committee of West China Hospital, Sichuan University, and all procedures were performed in compliance with the Declaration of Helsinki (Approval No. 201907V3, 1 July 2019). Before enrollment, all participants were informed of the ethical considerations related to the study and provided written informed consent. Patients with PC were eligible for inclusion if they met the following criteria: (1) pathologically confirmed pancreatic cancer; (2) no prior treatment before enrollment; and (3) no history of other primary malignancies. Patients were excluded if they had: (1) a history of previous cancer-related therapies; (2) incomplete medical records; and (3) concurrent inflammatory diseases. The control group consisted of patients with benign pancreatic diseases and healthy individuals. Benign pancreatic diseases included benign pancreatic tumors, chronic pancreatitis, and other non-malignant pancreatic disorders, while healthy controls had no clinical evidence of disease. The pathological diagnosis and histological grade of pancreatic cancer were performed by the Department of Pathology at West China Hospital. Tumor staging was determined according to the TNM classification based on the criteria of AJCC. Overall survival (OS) follow-up data were available for 39 of the 89 patients with PC and were obtained through outpatient visits or telephone interviews. The follow-up period ended in October 2023. Patients who were lost to follow-up were excluded from the survival analysis. The study design and workflow are illustrated in Figure S1.

### 2.2. Samples Collection

A total of 191 blood samples were collected in this study, including 89 patients with PC and 102 controls. The control cohort included 55 patients with benign pancreatic diseases (BDG), comprising chronic pancreatitis (*n* = 34), pancreatic neuroendocrine tumors (*n* = 4), intraductal papillary mucinous neoplasms (*n* = 8), pancreatic serous cystadenomas (*n* = 5), and solid pseudopapillary neoplasms (*n* = 4), together with 47 healthy controls (HC). All blood samples were collected prior to surgical resection, radiotherapy, or chemotherapy. Samples exhibiting hemolysis were excluded from the study. Fasting peripheral blood (5 mL) was collected from each participant into serum collection tubes and stored at 4 °C before being transported to the laboratory. Following centrifugation at 3000 rpm for 10 min at 4 °C, the serum was divided into two to four aliquots and stored at −80 °C until analysis. Additional clinicopathological data, including age, sex, histological grade, TNM stage, and serum levels of CA19-9, were obtained from the medical record management system of West China Hospital.

### 2.3. Measurement of Serum SAMD9 Levels

Serum SAMD9 levels were quantified using a commercially available ELISA kit (Cat. No. RX 2D2050186; Ruixinbio, Quanzhou, China) according to the manufacturer’s instructions. According to the instructions, add the standard, serum samples, and HRP-labeled detection antibody to the pre-coated microplate. Incubate at 37 °C for 60 min, then shake off the liquid from the wells, pat the plate dry, and wash 5 times. Add 100 μL of TMB substrate solution and incubate at 37 °C, protected from light, for 15 min. Finally, add 50 μL of stop solution and immediately measure the absorbance at 450 nm wavelength. SAMD9 concentrations were subsequently determined by comparing the absorbance values with those of the standard samples and calculating the concentrations based on the standard curve.

### 2.4. Public Data Sources

Pan-cancer analysis of *SAMD9* expression was performed using the SangerBox 3.0 platform [21]. TCGA, HPA, the UALCAN platform, GEO and GTEx databases were used to analyze the mRNA and protein expression levels of *SAMD9* [22,23], as well as the association between *SAMD9* expression levels and the clinicopathological features of patients with PC. For analyses integrating TCGA and GTEx samples, uniformly processed RNA-seq data were obtained from the UCSC Xena TCGA–GTEx Toil RNA-seq Recompute dataset.

### 2.5. Differential Expression Profiling and Pathway Enrichment Analysis

Patients were divided into high and low *SAMD9* expression groups according to the median *SAMD9* expression level. Cases with missing data for a specific clinical variable were excluded from the corresponding analysis, and no imputation was performed. The limma R package (version 3.66.0) was used to analyze differential gene expression in pancreatic cancer tissues. Genes meeting the thresholds of FDR-adjusted *p* < 0.05 and |logFC| > 1 were classified as differentially expressed genes (DEGs). GO and KEGG enrichment analyses were performed for the differentially expressed genes [24]. Gene set enrichment analysis (GSEA) was performed using the clusterProfiler package (version 4.18.3). All genes included in the differential expression analysis were ranked in descending order according to their logFC values. For duplicated gene symbols, the entry with the largest absolute logFC value was retained. Hallmark gene sets for *Homo sapiens* were obtained from the Molecular Signatures Database (MSigDB) using the msigdbr package (version 25.1.1) and were used as the reference gene sets. Pathway enrichment was evaluated using the normalized enrichment score (NES), and pathways with an adjusted *p* < 0.05 were considered significantly enriched.

### 2.6. Diagnostic and Prognostic Value Analysis

Diagnostic performance was assessed by ROC curve analysis using the pROC package in R (version 1.19.0.1), and quantified by the area under the curve (AUC). The diagnostic accuracy was classified according to AUC values as fail (0.50–0.60), poor (0.60–0.70), fair (0.70–0.80), good (0.80–0.90), or excellent (0.90–1.00). Kaplan–Meier analysis and Cox proportional hazards regression were used to investigate the prognostic relevance of *SAMD9* expression.

### 2.7. Statistical Analysis

GraphPad Prism 9.5 and R software (version 4.5.2) were used for statistical analyses. Depending on the type of data, Student’s *t*-test, one-way ANOVA, or the chi-square test was employed to analyze differences in gene expression, clinicopathological correlations, and prognostic outcomes. Differences between the areas under the ROC curves (AUCs) were compared using DeLong’s test. * *p* < 0.05, ** *p* <0.01, *** *p* < 0.001.

## 3. Results

### 3.1. Pan-Cancer Analysis and Expression of SAMD9 in Pancreatic Cancer

**Figure 1:**
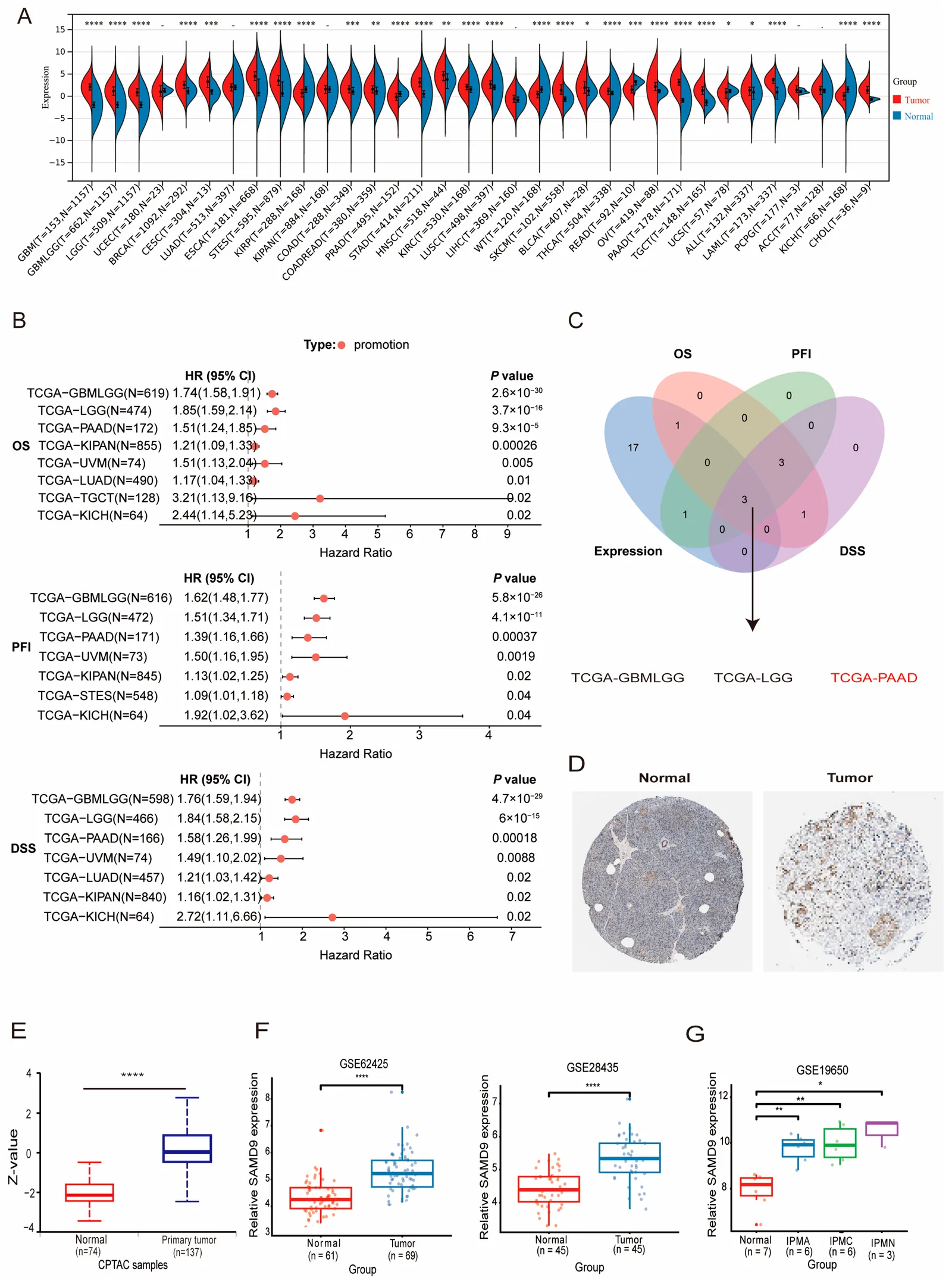
The expression levels of sterile alpha motif domain containing 9 (*SAMD9*) in pancreatic cancer (PC). (**A**) *SAMD9* expression in pan-cancer. (**B**) Univariate COX regression forest plot for OS, PFI, and PFS of *SAMD9*. (**C**) Venn diagram of cancer types with upregulated *SAMD9* expression and significant OS, PFI and DSS associations. (**D**) Immunohistochemical staining of SAMD9 in PC and normal samples from HPA. (**E**) Expression level of SAMD9 in PC and normal tissues based on the UALCAN platform. (**F**) Expression level of *SAMD9* in GSE62425 and GSE28435. (**G**) Expression level of *SAMD9* in GSE19650. * *p* < 0.05, ** *p* < 0.01, *** *p* < 0.001, **** *p* < 0.0001.

A comprehensive pan-cancer analysis of sterile alpha motif domain containing 9 (*SAMD9*) expression across 34 human tumor types was performed using the SangerBox 3.0 platform. *SAMD9* was significantly upregulated in 22 cancer types (Figure 1A). For these cancer types with significant differential expression, univariate Cox regression analysis was conducted to evaluate the association between *SAMD9* expression and patient prognosis, including overall survival (OS), progression-free interval (PFI), and progression-free survival (PFS) (Figure 1B). Significant prognostic associations were observed in GBMLGG (Glioma), LGG (Brain Lower Grade Glioma), PAAD (Pancreatic adenocarcinoma). Given the highly aggressive nature and poor prognosis of pancreatic cancer, subsequent analyses focused on the role of *SAMD9* in pancreatic cancer (PC) (Figure 1C). Immunohistochemical (IHC) images from the Human Protein Atlas (HPA) demonstrated elevated SAMD9 protein expression in pancreatic cancer tissues (Figure 1D). To further validate the differential expression of SAMD9 in PC, protein expression data from the UALCAN platform were analyzed, revealing significantly increased SAMD9 protein expression in PC tissues compared with normal pancreatic tissues (Figure 1E). In addition, analysis of the GEO datasets GSE28435 and GSE62425 further confirmed that *SAMD9* mRNA expression was significantly higher in tumor tissues than in normal pancreatic tissues (Figure 1F). Considering that PC develops progressively from precursor pancreatic lesions [25], we further assessed *SAMD9* expression in low-grade pancreatic lesions using data from the GSE19650 dataset. The results showed that *SAMD9* transcript levels were significantly increased in intraductal papillary mucinous neoplasms (IPMNs) compared with normal pancreatic tissues (Figure 1G).

### 3.2. Diagnostic and Prognostic Value of SAMD9 in Pancreatic Cancer

**Figure 2:**
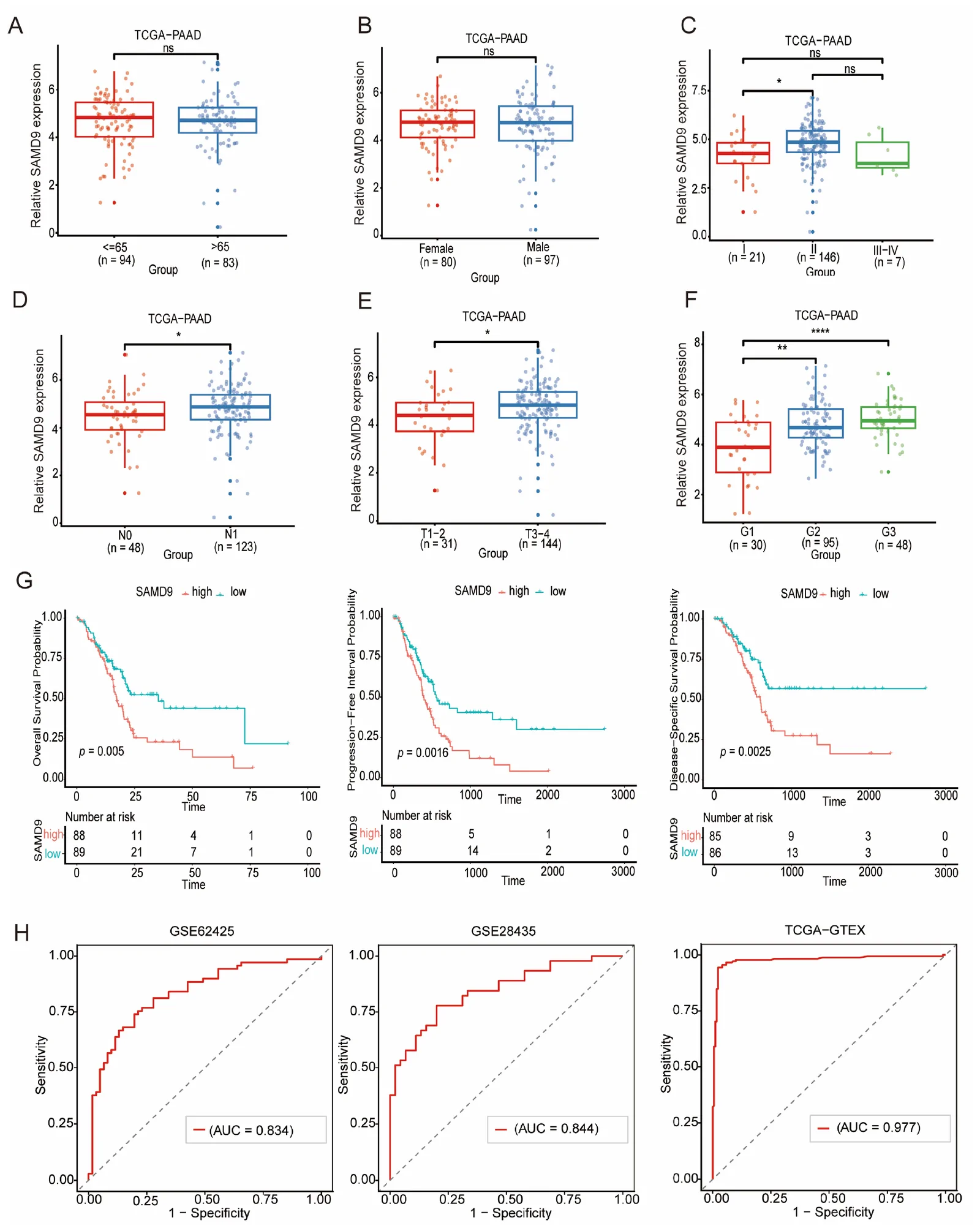
Clinicopathological associations and diagnostic and prognostic value of *SAMD9* in pancreatic cancer. (**A**–**F**) Expression of *SAMD9* across subgroups defined by age, gender, TNM stage, lymph node metastasis, tumor size and histological grades in PAAD. (**G**) Kaplan–Meier curves revealing that PAAD patients exhibiting high levels of *SAMD9* had poor overall survival, progression-free survival and disease-specific survival, compared to those with low expression in TCGA (left to right). (**H**) ROC curves of *SAMD9* expression in GEO cohorts and TCGA, GTEx. * *p* < 0.05, ** *p* < 0.01, **** *p* < 0.0001, ns, not significant.

Further analysis of the TCGA-PAAD dataset revealed significant associations between *SAMD9* expression and the clinicopathological characteristics of patients with pancreatic cancer. No significant differences in *SAMD9* expression were observed according to sex or age (Figure 2A,B). With respect to pathological stage, patients with stage II disease exhibited significantly higher *SAMD9* expression than those with stage I disease, whereas no significant differences were observed between stage III–IV and stage I or II disease (Figure 2C). In addition, *SAMD9* expression was significantly higher in patients with N1 stage than in those with N0 stage (Figure 2D), and was also significantly increased in T3–T4 tumors compared with T1–T2 tumors (Figure 2E). Regarding histological grade, *SAMD9* expression was significantly higher in both G2 and G3 tumors than in G1 tumors (Figure 2F). Kaplan–Meier survival analysis demonstrated that patients with high *SAMD9* expression had significantly shorter overall survival (OS), disease-specific survival (DSS), and progression-free interval (PFI) than those with low *SAMD9* expression (Figure 2G). Multivariate Cox regression analysis identified histological grade and high *SAMD9* expression as independent prognostic risk factors for overall survival in patients with PAAD (Table 1). To evaluate the diagnostic performance of *SAMD9*, receiver operating characteristic (ROC) curve analysis was performed. The area under the curve (AUC) values were 0.977 in the TCGA-GTEx cohort, 0.834 in the GSE62425 cohort, and 0.844 in the GSE28735 cohort, indicating good diagnostic performance of *SAMD9* for pancreatic cancer (Figure 2H).

### 3.3. Differential Gene Expression and Functional Enrichment Analysis

**Figure 3:**
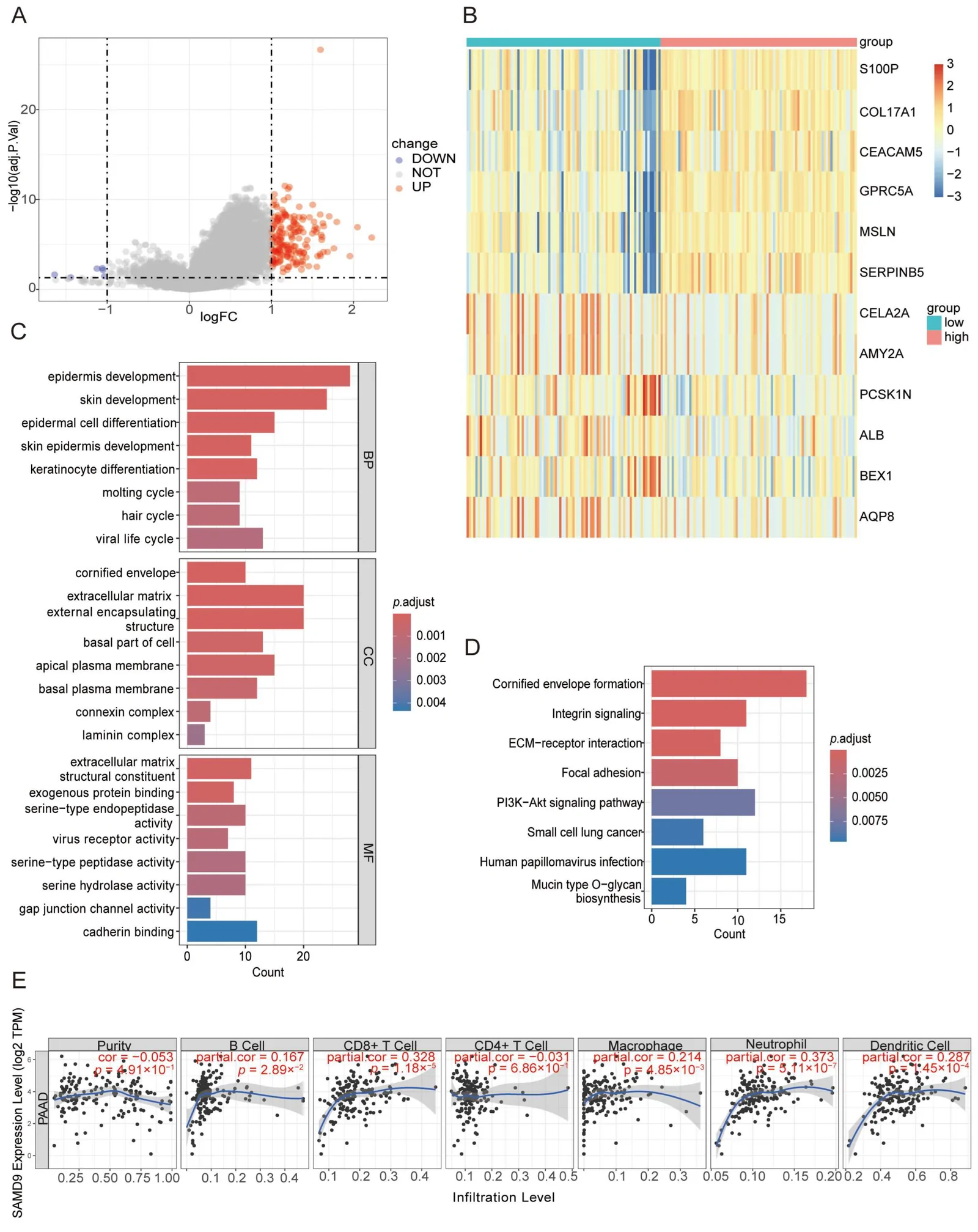
Differential gene expression, functional enrichment, and immune infiltration analyses associated with *SAMD9* expression in pancreatic cancer. (**A**) Volcano plot of DEGs in high *SAMD9* group in TCGA cohort compared with low *SAMD9* group. (**B**) Heatmap illustrating the top 6 upregulated and top 6 downregulated differentially expressed genes between the *SAMD9* high and low expression groups. (**C**) GO enrichment analysis of DEGs. (**D**) KEGG enrichment analysis of DEGs. (**E**) TIMER analysis of *SAMD9* expression and immune infiltration in pancreatic cancer.

**Figure 4:**
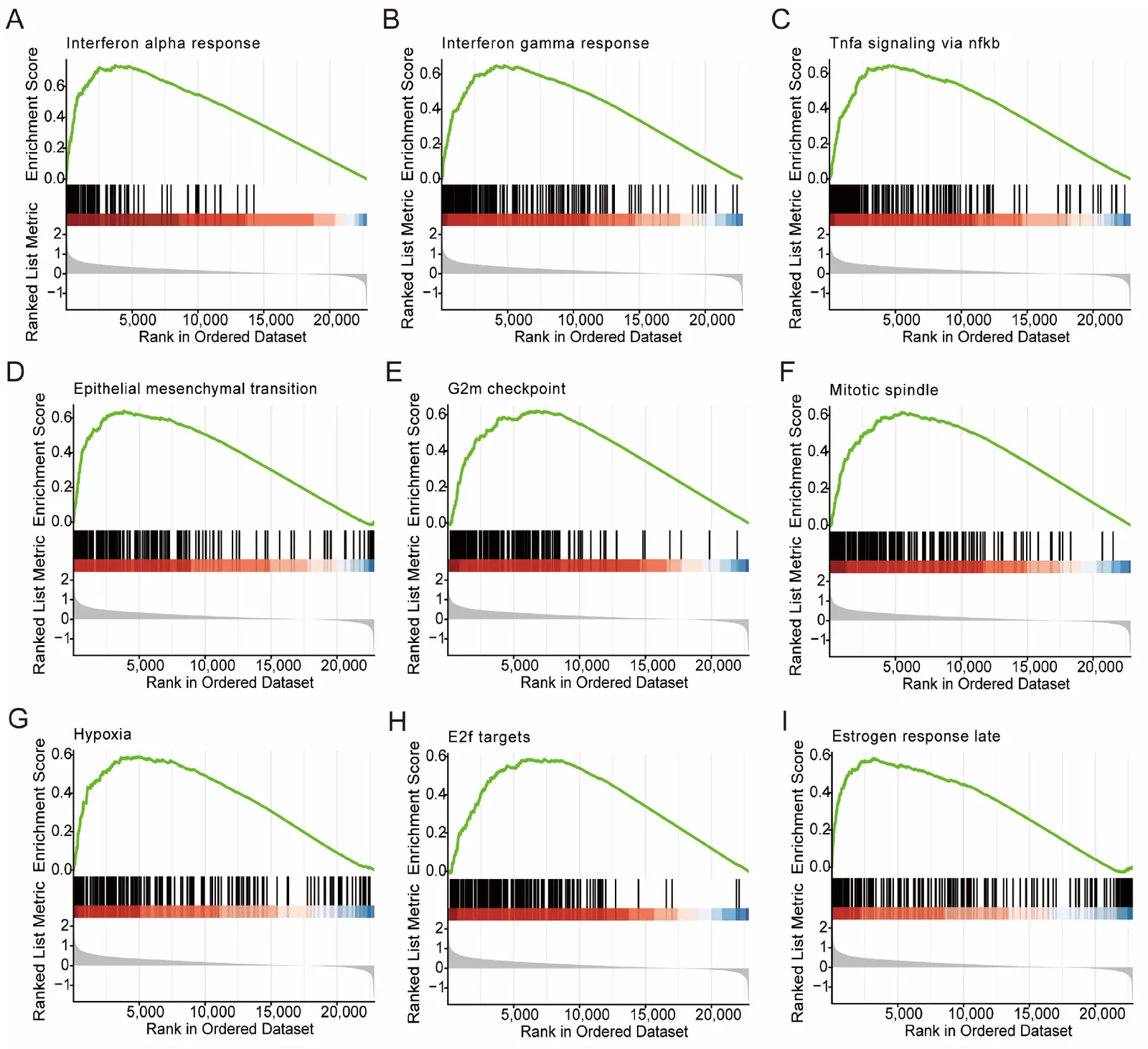
GSEA of biological pathways associated with *SAMD9* expression in pancreatic cancer (**A**–**I**). The green curve indicates the running enrichment score, and the black vertical lines indicate the positions of genes in the gene set within the ranked gene list. The red-to-blue color bar shows the ranking metric of differentially expressed genes between the SAMD9-high and SAMD9-low groups, where red indicates positive association with the SAMD9-high group and blue indicates positive association with the SAMD9-low group. The gray shaded area represents the ranked-list metric.

To investigate the potential molecular mechanisms of *SAMD9* in PC, patients were stratified into high and low *SAMD9* expression groups using the median expression level as the cutoff. Overall, differential expression analysis revealed 194 differentially expressed genes (DEGs), with 188 showing upregulated expression and 6 exhibiting downregulated expression (Figure 3A). The top 6 upregulated and downregulated DEGs are visualized in the heatmap (Figure 3B). To further explore the biological significance of the identified DEGs, GO enrichment analysis was performed. The results demonstrated that, in the Biological Process (BP) category, the DEGs were predominantly enriched in processes related to epidermis development, skin development, and epidermal cell differentiation. In the Cellular Component (CC) category, significant enrichment was observed in the cornified envelope, extracellular matrix, external encapsulating structure and basal part of cell. Molecular Function (MF) analysis revealed enrichment in extracellular matrix structural constituent, exogenous protein binding, serine-type endopeptidase activity and virus receptor activity (Figure 3C). KEGG enrichment analysis identified significant enrichment of the DEGs in Cornified envelope formation, Integrin signaling, ECM-receptor interaction, Focal adhesion and PI3K-AKT signaling pathway (Figure 3D). The biological pathways associated with *SAMD9* expression were further examined using Gene Set Enrichment Analysis (GSEA). The significantly enriched pathways were mainly involved in immune regulation, cell cycle regulation, and malignant phenotype progression, including Interferon Alpha Response, Interferon Gamma Response, TNF-α Signaling via NF-κB, G2M Checkpoint, Mitotic Spindle, E2F Targets, EMT, Hypoxia, and Estrogen Response Late (Figure 4A–I). Immune infiltration analysis based on the TIMER2.0 database was conducted to further examine the association of *SAMD9* expression with the tumor immune microenvironment [26]. Positive associations were observed between *SAMD9* expression and the infiltration of CD8^+^ T cells (partial correlation = 0.328, *p* < 0.001), macrophages (partial correlation = 0.214, *p* < 0.001), neutrophils (partial correlation = 0.373, *p* < 0.001), and dendritic cells (partial correlation = 0.287, *p* < 0.001). No significant association was observed with CD4^+^ T-cell infiltration or tumor purity (Figure 3E).

### 3.4. Clinical Significance and Diagnostic and Prognostic Value of SAMD9 in Pancreatic Cancer

**Figure 5:**
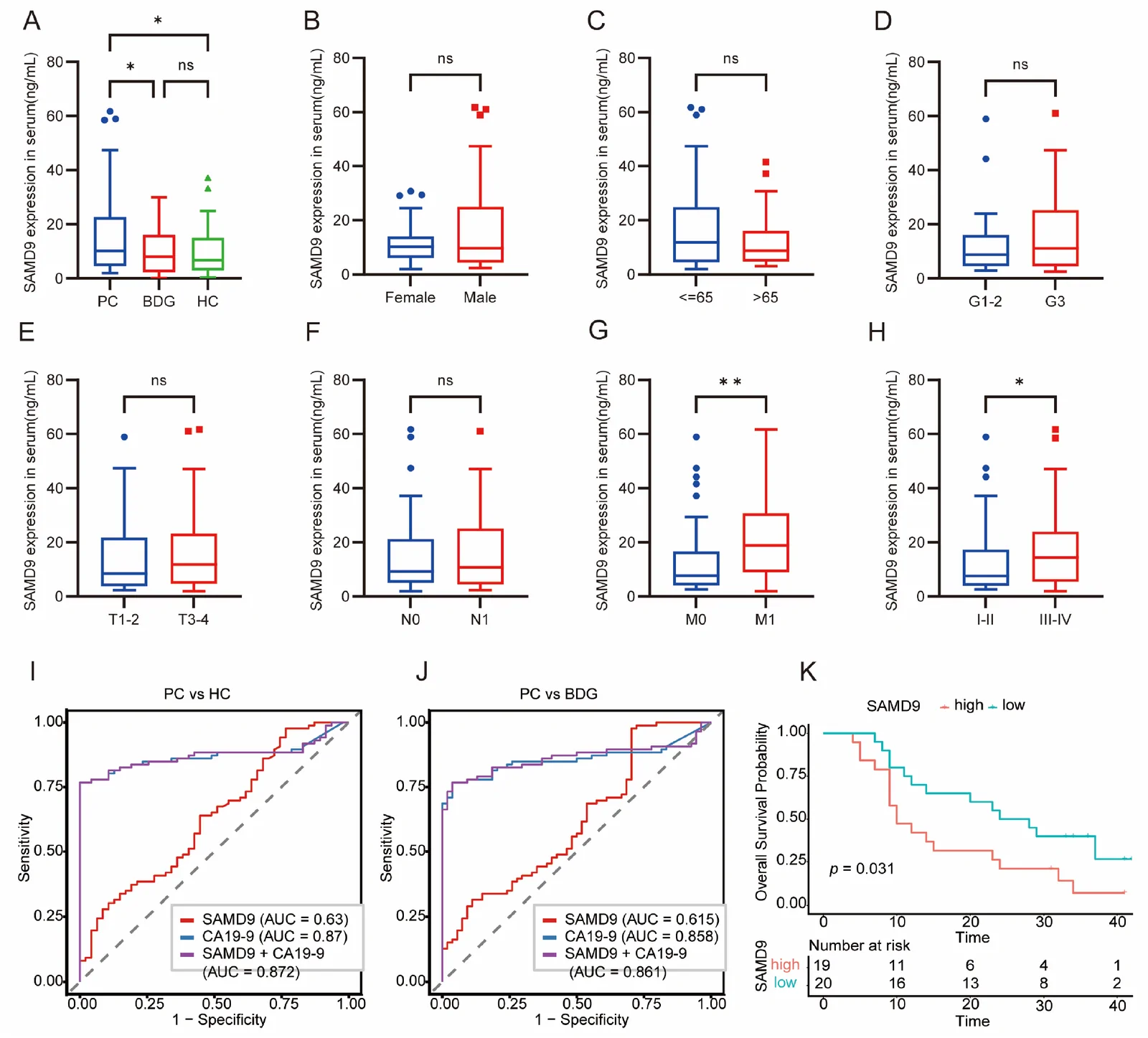
Serum SAMD9 expression and its clinical value in pancreatic cancer. (**A**) Serum SAMD9 levels among PC, BDG, and HC. (**B**–**H**) Association between serum SAMD9 levels and clinicopathological characteristics, including sex, age, histological grade, T stage, lymph node status, distant metastasis, and TNM stage. (**I**) ROC curve analysis of serum SAMD9 and CA19-9 for distinguishing PC from HC. (**J**) ROC curve analysis of serum SAMD9 and CA19-9 for distinguishing PC from BDG. (**K**) Kaplan–Meier overall survival analysis in patients with PC. * *p* < 0.05, ** *p* <0.01, ns, not significant.

A total of 191 participants were enrolled in this study, and their baseline characteristics are summarized in Table 2. Serum SAMD9 levels were significantly higher in patients with PC than in both HC and patients with BDG (*p* < 0.05), whereas no significant difference was observed between the HC and BDG groups (Figure 5A). To further examine the clinical significance of serum SAMD9, its association with the clinicopathological characteristics of patients with PC was analyzed. No significant differences in serum SAMD9 levels were observed according to sex or age (Figure 5B,C). Similarly, no significant differences were found between T1–T2 and T3–T4 tumors, lymph node invasion, or histological grade (Figure 5D–F). However, patients with stage III–IV disease exhibited significantly higher serum SAMD9 levels than those with stage I–II disease (Figure 5G). In addition, serum SAMD9 levels were significantly higher in patients with M1 than M0 (Figure 5H). To further investigate the clinical significance of serum SAMD9, patients with PC were divided into high and low SAMD9 groups according to the median serum SAMD9 concentration. The associations between serum SAMD9 levels and clinicopathological characteristics were evaluated using the χ^2^ test (Table 3). High serum SAMD9 levels were significantly associated with distant metastasis (*p* < 0.01) and advanced TNM stage (*p* < 0.01). In contrast, serum SAMD9 levels were not significantly associated with age, sex, smoking status, hypertension, diabetes, tumor invasion, lymph node metastasis, or histological grade (*p* > 0.05).

The diagnostic performance of serum SAMD9 was assessed using ROC curve analysis. When distinguishing patients with PC from HC, the AUC values for SAMD9, CA19-9 and SAMD9 + CA19-9 were 0.63, 0.87 and 0.872, respectively (Figure 5I). When differentiating patients with PC from those with benign pancreatic diseases (BDG), the AUC values for SAMD9, CA19-9 and SAMD9 + CA19-9 were 0.615, 0.858, and 0.861, respectively (Figure 5J). Comparison of the ROC curves using DeLong’s test showed that these differences were not statistically significant (*p* > 0.05), indicating that SAMD9 + CA19-9 provided limited incremental diagnostic value beyond CA19-9 alone. The sensitivity and specificity of SAMD9 and CA19-9 for PC detection are presented in Table 4. For stage I–II PC, serum SAMD9 showed limited diagnostic performance, with an AUC of 0.572 (sensitivity, 57.1%; specificity, 55.3%) versus HC and 0.550 (sensitivity, 61.9%; specificity, 46.3%) versus BDG. These findings indicate that SAMD9 alone has limited utility for identifying early-stage PC (Figure S2). Among the 39 patients with available survival data, 30 deaths occurred during follow-up, and the median follow-up duration was 36 months. Patients with available survival data were stratified into high and low serum SAMD9 groups according to the median serum SAMD9 concentration to further assess the prognostic value of serum SAMD9. Kaplan–Meier survival analysis demonstrated that patients in the high SAMD9 group (*n* = 19) had significantly shorter overall survival than those in the low SAMD9 group (*n* = 20) (*p* < 0.05) (Figure 5K). The prognostic analysis was exploratory and unadjusted.

## 4. Discussion

PC remains one of the most lethal malignancies worldwide, and reliable biomarkers for diagnosis and prognostic assessment are still lacking [27]. In the present study, we systematically evaluated the expression pattern, diagnostic value, and prognostic significance of SAMD9 in PC through integrated bioinformatics analyses and clinical validation. Genetic alterations of *SAMD9* were also identified in PC, although at a relatively low frequency, with mutations detected in only 2 of 175 cases (1.14%) in the TCGA-PAAD cohort. These alterations may potentially influence *SAMD9* expression. However, given their low frequency, the impact of *SAMD9* mutations on its diagnostic and prognostic value remains unclear and requires further investigation. In the present study, serum SAMD9 also showed diagnostic potential for distinguishing patients with PC from controls. However, in this cohort, its diagnostic performance was limited in patients with stage I–II PC. Unlike CA19-9, which is a well-established serum biomarker for PC, SAMD9 may reflect different biological aspects of tumor development and the tumor microenvironment. Therefore, the two biomarkers may provide partially complementary diagnostic information. However, the improvement in diagnostic performance achieved by combining SAMD9 with CA19-9 was modest, and its clinical utility requires further validation in larger independent cohorts.

Accumulating evidence has demonstrated that *SAMD9* participates in the regulation of multiple cancer-related signaling pathways. In esophageal squamous cell carcinoma, *SAMD9* promotes cancer stemness, EMT, angiogenesis, and postoperative recurrence through activation of the Wnt/β-catenin pathway [28], whereas in glioma it has been implicated in tumor progression via PI3K/AKT signaling [29]. Consistent with these observations, our study demonstrated that elevated *SAMD9* expression was associated with poor prognosis in patients with PC. To gain insight into the biological role of *SAMD9* in PC, we performed functional enrichment analyses. GO analysis revealed enrichment in terms related to epidermal differentiation and extracellular matrix components, whereas KEGG analysis identified pathways including focal adhesion, ECM–receptor interaction, integrin signaling, and PI3K–AKT signaling. These findings suggest that *SAMD9*-associated gene expression may be linked to extracellular matrix and cell adhesion-related biological processes. In addition, GSEA demonstrated significant enrichment of interferon-related signaling, TNF-α signaling via NF-κB, EMT, and hypoxia, further indicating that *SAMD9* expression is associated with immune-related and malignant progression-related molecular features. Consistent with these findings, TIMER analysis further revealed positive correlations between *SAMD9* expression and the infiltration of CD8^+^ T cells, macrophages, neutrophils, dendritic cells, and B cells. Together, these findings suggest a potential association between *SAMD9* expression and the tumor immune microenvironment of PC [30]. However, these bioinformatic observations do not establish a direct functional or causal role of *SAMD9* in these biological processes.

Although this study comprehensively evaluated the expression pattern and clinical significance of *SAMD9* in PC through integrated bioinformatics analyses and clinical validation, several limitations should be acknowledged. First, this was a retrospective study based primarily on public databases, which may be subject to unavoidable selection bias. Differences in age and sex among the clinical groups may also have introduced potential confounding effects; therefore, the diagnostic performance observed in the present cohort should be interpreted with caution. Second, the relatively small clinical sample size limits the generalizability of our findings, and the diagnostic and prognostic value of SAMD9 requires further validation in larger, stage-stratified, and multicenter cohorts. Third, given the limited sample size and the absence of multivariable adjustment, the prognostic findings from the clinical cohort should be interpreted with caution and require validation in larger cohorts. Finally, the enrichment and immune infiltration analyses indicate associations. The molecular mechanisms underlying the role of *SAMD9* in PC were not experimentally investigated, and further in vitro and in vivo studies are warranted to clarify its biological functions.

## 5. Conclusions

In conclusion, the present study demonstrated that SAMD9 is significantly upregulated in PC and is associated with unfavorable prognosis. These findings support the potential prognostic value of SAMD9 in PC, while its diagnostic utility, particularly as diagnostic biomarker, appears limited and warrants further investigation.

## Figures and Tables

**Table 1:** Univariate and multivariate Cox analyses for predicting OS of PAAD in the TCGA database.

Clinical Variable	Univariate		Multivariate	
	HR (95% CI)	*p*-Value	HR (95% CI)	*p*-Value
*SAMD9* expression (high vs. low)	1.805 (1.187–2.743)	0.006	1.704 (1.110–2.617)	0.015
Age (>65 vs. ≤65)	1.307 (0.867–1.969)	0.201		
Gender (Male vs. Female)	0.823 (0.548–1.238)	0.350		
TNM stage (III–IV vs. I–II)	0.801 (0.253–2.539)	0.706		
Grade (G2–3 vs. G1)	2.190 (1.153–4.157)	0.017	1.993 (1.045–3.799)	0.036

**Table 2:** Clinicopathological characteristics of patients with pancreatic cancer and benign pancreatic diseases.

Characteristic	Pancreatic Cancer (*n* = 89)	Benign Pancreatic Tumor (*n* = 21)	Chronic Pancreatitis (*n* = 34)	*p*-Value
Age, year				<0.001
<65	50 (56.2%)	19 (90.5%)	32 (94.1%)	
≥65	39 (43.8%)	2 (9.5%)	2 (5.9%)	
Sex, *n* (%)				0.003
Male	60 (67.4%)	7 (33.3%)	26 (76.5%)	
Female	29 (32.6%)	14 (66.7%)	8 (23.5%)	
BMI	21.94 ± 3.07	22.17 ± 4.03	21.69 ± 2.90	0.936
Smoke, *n* (%)				0.074
Yes	31 (34.8%)	3 (14.3%)	15 (44.1%)	
No	58 (65.2%)	18 (85.7%)	19 (55.9%)	
Drink, *n* (%)				0.146
Yes	21 (23.6%)	2 (9.5%)	11 (32.4%)	
No	68 (76.4%)	19 (90.5%)	23 (67.6%)	
Hypertension, *n* (%)				0.919
Yes	21 (23.6%)	4 (19.0%)	7 (20.6%)	
No	68 (76.4%)	17 (81.0%)	27 (79.4%)	
Diabetes, *n* (%)				0.367
Yes	13 (14.6%)	2 (9.5%)	8 (23.5%)	
No	76 (85.4%)	19 (90.5%)	26 (76.5%)	
T stage, *n* (%)				
T1	3 (3.4%)			
T2	33 (37.1%)			
T3	17 (19.1%)			
T4	36 (40.4%)			
Lymph node invasion, *n* (%)				
N0	53 (59.6%)			
N1	36 (40.4%)			
Metastasis, *n* (%)				
M0	62 (69.7%)			
M1	27 (30.3%)			
TNM stage, *n* (%)				
I	23 (25.8%)			
II	19 (21.3%)			
III	20 (22.5%)			
IV	27 (30.3%)			
Tumor differentiation, *n* (%)				
Missing	26 (29.2%)			
Well	2 (2.2%)			
Moderate	25 (28.1%)			
Poor	36 (40.4%)			

**Table 3:** Clinical and pathological characteristics of patients based on SAMD9 expression.

Characteristics	Number	SAMD9 Low	SAMD9 High	χ^2^	*p*-Value
Age, year					
<65	50	21 (46.7%)	29 (65.9%)	3.346	0.106
≥65	39	24 (53.3%)	15 (34.1%)		
Sex, *n* (%)					
Female	29	15 (33.3%)	14 (31.8%)	0.023	1.000
Male	60	30 (66.7%)	30 (68.2%)		
Smoke, *n* (%)					
No	58	29 (64.4%)	29 (65.9%)	0.021	1.000
Yes	31	16 (35.6%)	15 (34.1%)		
Drink, *n* (%)					
No	68	35 (77.8%)	33 (75.0%)	0.095	0.953
Yes	21	10 (22.2%)	11 (25.0%)		
Hypertension, *n* (%)					
No	68	34 (75.6%)	34 (77.3%)	0.036	1.000
Yes	21	11 (24.4%)	10 (22.7%)		
Diabetes, *n* (%)					
No	76	40 (88.9)	36 (81.8%)	0.892	0.519
Yes	13	5 (11.1%)	8 (18.2%)		
T stage, *n* (%)					
T1–2	36	22 (48.9%)	14 (31.8%)	2.691	0.154
T3–4	53	23 (51.1%)	30 (68.2%)		
Lymph node invasion, *n* (%)					
Negative	53	28 (62.2%)	25 (56.8%)	0.27	0.762
Positive	36	17 (37.8%)	19 (43.2%)		
Metastasis, *n* (%)					
Negative	62	38 (84.4%)	24 (54.5%)	9.411	0.005
Positive	27	7 (15.6%)	20 (45.5%)		
TNM stage, *n* (%)					
I–II	42	28 (62.2%)	14 (31.8%)	8.252	0.008
III–IV	47	17 (37.8%)	30 (68.2%)		
Tumor differentiation, *n* (%)					
Well-Moderate	27	15 (46.9%)	12 (38.7%)	0.429	0.689
Poor	37	17 (53.1%)	19 (61.3%)		
Missing	26				

**Table 4:** Diagnostic performance of SAMD9 alone and in combination with CA19-9 for the detection of PC.

Biomarkers	Cut-Off	AUC	95% CI	Sensitivity (%)	Specificity (%)
PC vs. HC					
SAMD9	7.297	0.630	0.531–0.73	64%	55.3%
CA19-9	21.65	0.870	0.807–0.934	80.2%	89.4%
SAMD9 + CA19-9	—	0.872	0.809–0.935	81.4%	89.4%
PC vs. BDG					
SAMD9	6.187	0.615	0.518–0.712	68.6%	46.3%
CA19-9	48.45	0.858	0.793–0.923	76.7%	96.3%
SAMD9 + CA19-9	—	0.861	0.797–0.926	76.7%	96.3%

## Data Availability

The dataset generated for this study is available on request to the corresponding author.

## Supplementary Materials

The following supporting information can be downloaded at: https://www.mdpi.com/article/10.3390/cancers18172781/s1, Figure S1. Flowchart of the study design; Figure S2. Diagnostic performance of serum SAMD9 in stage I–II pancreatic cancer. (A) ROC curve analysis of serum SAMD9 and CA19-9 for distinguishing PC I-II from HC. (B) ROC curve analysis of serum SAMD9 and CA19-9 for distinguishing PC I-II from BDG.
