# Supplementary material for: Serum SAMD9 as a Potential Prognostic Biomarker for Pancreatic Cancer
Source: Cancers (Basel). 2026 Aug 27;18(17):2781. doi: 10.3390/cancers18172781 (PMC13565438; doi:10.3390/cancers18172781)

Supplementary Figure

Figure S1 Flowchart of the study design.

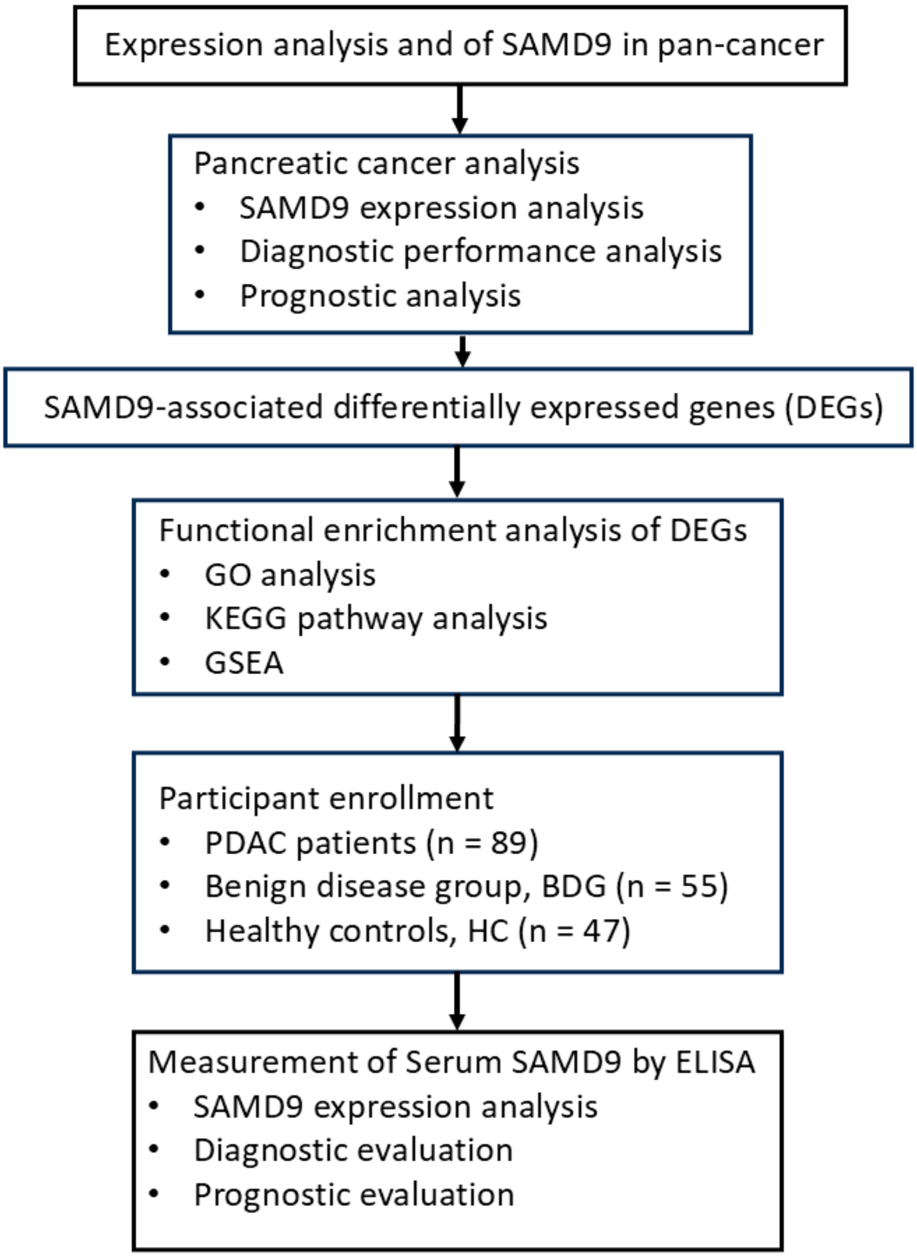

Figure S2 Diagnostic performance of serum SAMD9 in stage I–II pancreatic cancer.

（A）ROC curve analysis of serum SAMD9 and CA19-9 for distinguishing PC I-II from HC. (B) ROC curve analysis of serum SAMD9 and CA19-9 for distinguishing PC I-II from BDG.

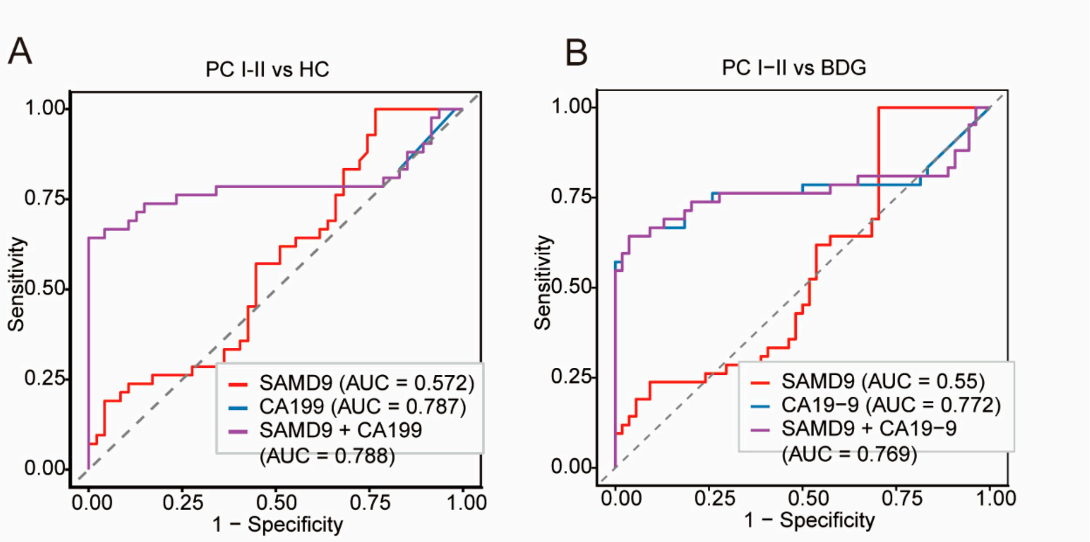

Supplement: Supplementary file 1 [file cancers-18-02781-s001.zip › cancers-4487186-supplementary.pdf]
